# Distinct pathophysiological cytokine profiles for discrimination between autoimmune pancreatitis, chronic pancreatitis, and pancreatic ductal adenocarcinoma

**DOI:** 10.1186/s12967-017-1227-3

**Published:** 2017-06-02

**Authors:** Sahar Ghassem-Zadeh, Matthias M. Gaida, Szilard Szanyi, Hans Acha-Orbea, Jean-Louis Frossard, Ulf Hinz, Thilo Hackert, Oliver Strobel, Klaus Felix

**Affiliations:** 1 Department of General, Visceral and Transplantation Surgery, University Hospital Heidelberg, Im Neuenheimer Feld 110, 69120 Heidelberg, Germany; 2 Department of Biochemistry, University of Lausanne, Lausanne, Switzerland; 3 Institute of Pathology, University of Heidelberg, Heidelberg, Germany; 4 Department of Medical Specialties, Gastroenterology and Hepatology Division, University Hospitals and Faculty of Medicine of Geneva, Geneva, Switzerland

**Keywords:** Autoimmune pancreatitis type 1 and type 2, Chronic pancreatitis, Pancreatic ductal adenocarcinoma, Cytokines

## Abstract

**Background:**

Discriminating between autoimmune pancreatitis (AIP), chronic pancreatitis (CP), and pancreatic ductal adenocarcinoma (PDAC) can be challenging. In this retrospective study, levels of serum and tissue cytokines were analyzed as part of the clinical strategy for the preoperative differentiation between AIP and PDAC. The identification of differential cytokine profiles may help to prevent unnecessary surgical resection and allow optimal treatment of these pathologies.

**Methods:**

To compare the cytokine profiles of AIP, CP, and PDAC patients, serum and pancreatic tissue homogenates were subjected to multiplex analysis of 17 inflammatory mediators. In total, serum from 73 patients, composed of 29 AIP (14 AIP-1 and 15 AIP-2), 17 CP, and 27 PDAC, and pancreatic tissue from 36 patients, including 12 AIP (six AIP-1 and six AIP-2), 12 CP, and 12 PDAC, were analyzed.

**Results:**

Comparing AIP and PDAC patients’ serum, significantly higher concentrations were found in AIP for interleukins IL-1β, IL-7, IL-13, and granulocyte colony-stimulating factor (G-CSF). G-CSF also allowed discrimination of AIP from CP. Furthermore, once AIP was divided into subtypes, significantly higher serum levels for IL-7 and G-CSF were measured in both subtypes of AIP and in AIP-2 for IL-1β when compared to PDAC. G-CSF and TNF-α were also significantly differentially expressed in tissue homogenates between AIP-2 and PDAC.

**Conclusions:**

The cytokines IL-1β, IL-7, and G-CSF can be routinely measured in patients’ serum, providing an elegant and non-invasive approach for differential diagnosis. G-CSF is a good candidate to supplement the currently known serum markers in predictive tests for AIP and represents a basis for a combined blood test to differentiate AIP and particularly AIP-2 from PDAC, enhancing the possibility of appropriate treatment.

**Electronic supplementary material:**

The online version of this article (doi:10.1186/s12967-017-1227-3) contains supplementary material, which is available to authorized users.

## Background

Autoimmune pancreatitis (AIP) is an immune-mediated fibro-inflammatory form of pancreatitis that has a unique histopathologic pattern characterized by periductal lymphoplasmocytic infiltration, storiform fibrosis, and obliterative phlebitis, which makes it distinguishable from other pancreas diseases [1, 2]. AIP, due to its immunological etiology, is the only type of pancreatitis that is responsive to steroid treatment [3, 4]. Nowadays, two types of AIP exist, defined by distinct histology and clinical profiles [5]. Furthermore there are differences in age of appearance [1, 2], sex ratio [1, 6–8], geographic distribution [5], and histological, immunological, and imaging features [2, 6–10].

AIP type 1 (AIP-1) (the most common type in Asia), also named lymphoplasmocytic sclerosing pancreatitis (LPSP) because of its histological features, is associated with elevated IgG4 serum levels and IgG4-positive cell infiltration, an increased number of various autoantibodies [2, 6, 9], and involvement of other organs, such as peritoneum, biliary tract etc., besides the pancreas, reflecting a systemic disease [11].

AIP type 2 (AIP-2), called idiopathic duct-centric pancreatitis (IDCP), is recognized by its specific histological feature: granulocytic epithelial lesions (GEL) [1, 2, 6, 12]. These lesions consist of a focal disruption and destruction of the duct epithelium caused by the invasion of neutrophilic granulocytes, which makes some people call it AIP with GEL. It is likely that deregulated cytokines and other transcription factors are the driving force by which neutrophils are recruited to the ductal and acinar cells with subsequent destruction. This latter subtype is more common in Western countries [5].

The etiology and pathophysiological mechanisms of AIP remain unknown but several findings suggest that an autoimmune mechanism might be involved, mainly because AIP is associated with hypergammaglobulinemia [2, 13] and increased levels of IgG4 in AIP-1 [2, 14, 15]. Additionally, high titers of circulating immune complexes, an elevated number of regulatory T cells (Tregs) in the tissue and blood of affected individuals [16, 17], and the presence of autoantibodies support the hypothesis of an autoimmune mechanism [18–20]. Finally, common association with other autoimmune diseases [11] and positive response to steroid therapy [3, 4] strengthen this theory. Cytokines are key mediators of innate and adaptive immunity. They regulate a large spectrum of processes including antigen presentation, bone marrow differentiation, and cellular activation [21]. Due to their implication in various pathologies including cancer and inflammatory diseases, expression of different cytokines has been assessed in order to explain the inflammatory process involved in AIP [22–24].

Most autoimmune diseases are known to have a Th1-predominant cytokine expression. However, in AIP both Th1 and Th2 cytokines have been described. Zen et al. showed overexpression of Th2 and regulatory cytokines in the tissue of patients with autoimmune pancreato-cholangitis [25], while Okazaki et al. [20] demonstrated an increase of Th1 cytokines in the peripheral blood of patients suffering from AIP. However, the lack of differentiation between AIP-1 and AIP-2 among AIP patients in the currently published data makes the interpretation of these studies very difficult.

Distinction between AIP subtypes from pancreatic ductal adenocarcinoma (PDAC) is difficult and clinically relevant since they require different treatment. Nowadays, different tools such as serological markers and imaging features can help, but still are not sufficient to discriminate accurately the diseases [26]. Currently, about 2.2 to 3.7% of patients undergoing surgery for suspected pancreatic cancer turn out to have AIP [27–29]. Furthermore, in an international multicenter survey, it was shown that 60% (123 of 204) and 78% (50 of 64) of AIP-1 and AIP-2 patients, respectively, were evaluated retrospectively from cases resected on suspicion of pancreatic cancer [5]. The need for new diagnostic tools is crucial to decrease these numbers.

The aim of the present investigation was to assess and compare expression profiles of different cytokines in serum and pancreatic tissue of both AIP subtypes and compare their levels with pancreatic cancer (PDAC) and chronic pancreatitis (CP).

## Methods

### Patients and samples

Banked serum (drawn upon admission or prior to surgery) and pancreatic tissue samples (obtained during surgery) were collected between 2001 and 2009, in the Biobank of the European Pancreas Center, Department of Surgery, Heidelberg University Hospital, Heidelberg. All sera were stored at −80 °C. Age, pathologic diagnosis, and serological analyses at the time of sample acquisition were obtained for all groups. Prior to analysis, all pancreatic tissues were reviewed by a pathologist to confirm disease diagnosis (AIP-1, AIP-2, CP, and PDAC).

#### Serum cytokine multiplexing

Sera included specimens from 29 AIP patients. Among them, 23 underwent pancreatic surgery, and histology confirmed the disease. Six patients were diagnosed according to the HISORt Mayo Clinic criteria [30] and by the effectiveness of steroid treatment. The CP and PDAC groups consisted of 17 and 27 serum samples, respectively (Table 1a).

**Table 1:** Patient data on samples used for serum and tissue extracts cytokine evaluation approaches

	Total	AIP-1	AIP-2	CP	PDAC
a Serum cytokine multiplexing					
Patients [n]	73	14	15	17	27
Male [n] (%)	50 (68.5)	14 (100)	11 (73.3)	162 (70.6)	13 (48.1)
Median age (IQR)	58.0 (21)	56.5 (21.3)	44.0 (25)	55.0 (16.5)	61.0 (13)
b Pancreatic tissue homogenate cytokine multiplexing					
Patients [n]	36	6	6	12	12
Male [n] (%)	28 (77.8)	6 (100)	5 (83.3)	8 (66.7)	9 (75)
Median age (IQR)	54.0 (21)	54.0 (12.0)	40.5 (21.25)	51.0 (12.5)	69.5 (24)

*IQR* inter quartile range

#### Pancreatic tissue homogenate cytokine multiplexing

The total of 36 pancreatic tissue samples investigated in this approach was composed of 12 AIP, 12 CP, and 12 PDAC. Patients’ data are presented in Table 1b.

### Multiplex cytokine analysis

A multiplex assay for simultaneous quantitative determination of proteins in diverse and complex biofluids was applied to assess concentrations of the selected cytokines in serum and tissue samples. The analysis was performed using the Bio-Plex Pro Human Cytokine 17-Plex panel (Bio-Rad Laboratories GmbH, Munich, Germany), a kit composed of 17 determinants. The following cytokines were simultaneously detected and standard curve range and limit of detection (LOD) all expressed in pg/ml are shown in parenthesis: IL-1β (0.34–4.991; LOD 0.06), IL-2 (2.27–9.531; LOD 0.62), IL-4 (0.22–3.207; LOD 0.15), IL-5 (3.25–13.069; LOD 2.3), IL-6 (1.41–23.178; LOD 0.61), IL-7 (0.68–11.063; LOD 0.66), IL-8 (1.38–2.183; 0.95), IL-10 (3.39–5.521; LOD 0.22), IL-12 (2.35–38.575; LOD 0.29), IL-13 (0.36–5.908; LOD 0.19), IL-17 (2.00–32.716; LOD 0.68), G-CSF (1.73–28.267; LOD 1.47), GM-CSF (2.45–10.848; LOD 1.47), IFN-γ (2.57–14.845; LOD 0.96), MCP-1 (3.72–55.124; LOD 2.65), MIP-1β (1.134–4.543; LOD 1.06), and TNF-α (3.18–52.115; LOD 1.88). A series of calibrators was analyzed with the patient samples to convert the fluorescence ratio to international units per milliliter. Concentration of each analyte was obtained by interpolating fluorescence intensity to eight-point dilution standard curve supplied by the kit. Values that were outside the standard curve range were calculated by the Brendan Scientific weighted five parameter fit algorithm as best estimation of the analyte concentrations within the samples and were obtained by extrapolation beyond the dynamic range of the standard curve and calculated by the Bio-Plex™ software (Bio-Plex Manager version 6.1 BioRad). All serum samples were measured in triplicate.”

Multiplexing was performed according to the manufacturer’s instruction manual. Briefly, for cytokine determination in serum, magnetic beads coated with antibodies against the examined antigens were mixed with 200 μl of each 1 : 4 v/v diluted patient sample (50 µl serum and 150 µl dilution buffer, Bio-Rad) and then incubated for 30 min. After a wash cycle followed by the addition of a detection antibody, another 30 min incubation, and a second wash cycle, streptavidin-PE was added to the beads. With the third wash cycle the excess conjugate was removed, and the bead mixture was analyzed on a Bio-Rad Bio-Plex 200 system.

For cytokine determination in pancreatic tissue, 60–80 mg of frozen tissue samples was cut with a cryomicrotome into 9 μm-thick slices (Leica CM 3050 S Leica Biosystems, Nussloch, Germany), collected in 15 ml polypropylene tubes, and mixed with 500 µl of lysis buffer (Bio-Rad). Each tube was vigorously vortexed then shock-frozen in liquid nitrogen and stored overnight at −80 °C. The day after, using an ultrasonic homogenizer (SonoPuls mini20 Bandelin^®^, Berlin, Germany), the suspensions were subjected to a 30 s sonication step on ice (ampl. 80%, 0.99 kJ) and subsequently centrifuged at 16,000×*g* for 10 min. Supernatants were collected and divided into aliquots, and the total protein concentration was determined using a Pierce BCA assay (Thermo, Rockford, IL, USA). For multiplexing, the supernatants were adjusted with a dilution buffer (Bio-Rad) to a total protein concentration of 600 μg/ml and analyzed using the Bio-Plex Pro Human Cytokine 17-Plex panel kit (Bio-Rad) according to the manufacturer’s protocol as described above.

### Statistical analyses

Statistical analyses were performed using GraphPad Prism software (version 5; La Jolla, CA, USA) and IBM SPSS Statistics version 22 (IBM Corp, Armonk, NY, USA). As variables were not normally distributed, the nonparametric Mann–Whitney test was used to assess the significant differences in multiplexing assays of serum and tissue extracts. The quantitative variables are graphically presented as box-and-whisker plots. Values of p ≤ 0.05 were considered to be significant. All tests were used two-sided.

Receiver operating characteristic (ROC) curves were calculated to analyze the test performance of serum cytokines G-CSF and IL-7 for predicting AIP using SAS software (release 9.4, SAS Institute, Inc., Cary, NC, USA). Logistic regression analysis was performed to generate sensitivity, specificity, and area under curve (AUC) values. Youden’s J statistic was used to select the optimal predicted probability cut-off.

## Results

### Serum cytokine profiling

All the comparisons on different cytokines were executed twice. At first, evaluations were performed between all AIP vs PDAC and CP patients and the reference category was always AIP. The comparison of serum cytokine expression levels in the three different groups is summarized in Table 2. The statistical analysis of serum comparing AIP to PDAC revealed a significantly higher concentration in AIP patients for IL-1β (p = 0.0221), for IL-7 (p = 0.0003), for IL-13 (p = 0.0337), and for G-CSF (p = 0.0105). The G-CSF levels in AIP (median 14.23 pg/ml) were also significantly discriminatory between AIP and CP (1.47 pg/ml) (p = 0.0006).

**Table 2:** Comparison of cytokine levels in serum from: AIP, CP and PDAC patients

Cytokine	Compared groups 1st group vs 2nd group	Median conc. (pg/ml) of 1st group/IQR/n	Median conc. (pg/ml) of 2nd group/IQR/n	p-value
IL-1β	AIP vs CP AIP vs PDAC	0.58/0.86/29 0.58/0.86/29	0.39/1.3/17 0.39/0.55/27	0.5193 *0.0221*
IL-6	AIP vs CP AIP vs PDAC	12.58/18.76/28 12.58/18.76/28	1.44/25.63/17 10.89/22.42/27	0.1399 0.4435
IL-7	AIP vs CP AIP vs PDAC	13.70/8.22/29 13.70/8.22/29	8.72/13.04/17 8.27/3.65/27	0.1386 *0.0003*
IL-8	AIP vs CP AIP vs PDAC	22.64/13.05/29 22.64/13.05/29	17.17/23.77/17 21.56/26.87/27	0.2410 0.4806
IL-10	AIP vs CP AIP vs PDAC	2.68/12.91/28 2.68/12.91/28	0.40/12.10/13 4.09/9.59/21	0.0792 0.9919
IL-13	AIP vs CP AIP vs PDAC	0.32/0.70/27 0.32/0.70/27	0.32/1.95/17 0.13/0.28/26	0.5025 *0.0337*
IL-17	AIP vs CP AIP vs PDAC	7.44/19.27/29 7.44/19.27/29	0.03/10.35/17 4.32/19.31/27	0.1239 0.5937
G-CSF	AIP vs CP AIP vs PDAC	14.23/16.85/29 14.23/16.85/29	1.47/3.93/17 5.23/13.82/27	*0.0006* *0.0105*
MCP-1	AIP vs CP AIP vs PDAC	56.35/65.13/29 56.35/65.13/29	10.61/97.51/17 43.41/104.96/27	0.0738 0.3890
MIP-1β	AIP vs CP AIP vs PDAC	130.02/65.10/29 130.02/65.10/29	96.66/62.28/17 122.72/83.47/27	0.0837 0.8956

Italic values indicate significance of p value (p < 0.05)

*IQR* interquartile range, *n* number of observations

**Fig. 1:**
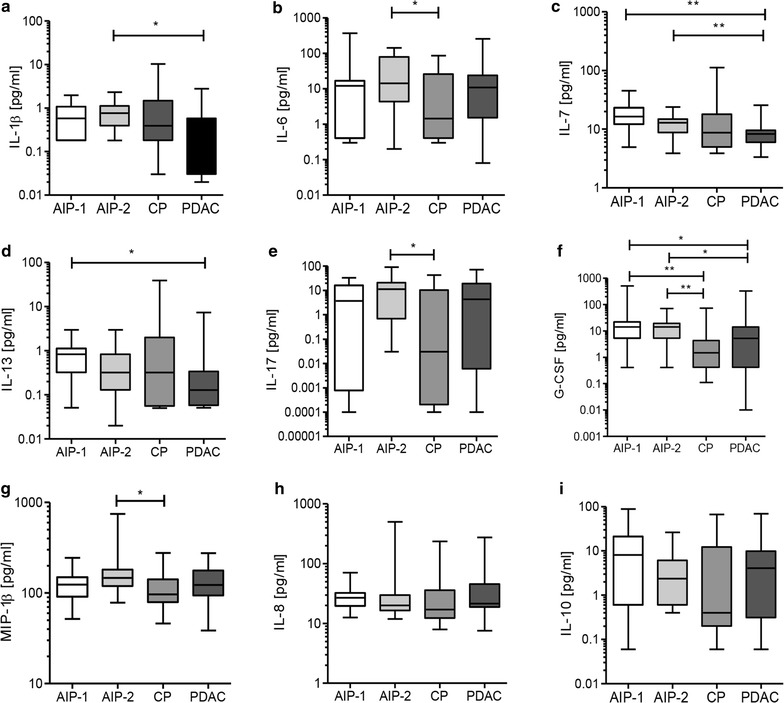
Serum levels and distribution range of the most differentially expressed cytokines (**a**–**i**) from AIP-1, AIP-2, CP, and PDAC patients. Differences were considered statistically significant when the p value was less than 0.05 and are marked with an *asterisk*: *p < 0.05, **p < 0.01

Subsequently, comparisons were performed among the AIP-1, AIP-2, PDAC, and CP groups using AIP-1 and AIP-2 separately as reference categories. When AIP-1, AIP-2, CP, and PDAC were compared and AIP-1 was used as a reference category, significantly higher concentrations of IL-7 (p = 0.0012), IL-13 (p = 0.0162), and G-CSF (p = 0.0425) were found in AIP-1 compared to PDAC. Also, a significantly higher level of G-CSF (p = 0.0039) in AIP-1 compared to CP was noticed. When we compared AIP-2 with PDAC, we found significantly higher levels of IL-1β (p = 0.0217), IL-7 (p = 0.005), and G-CSF (p = 0.032) in AIP-2. Comparing AIP-2 with CP, significantly higher levels in AIP-2 were found for IL-6 (p = 0.0361), for IL-17 (p = 0.0377), and G-CSF (p = 0.0034). The range of cytokine distribution in the analyzed groups is presented in Fig. 1a–i and the median concentrations with the IQR of analyzed samples are summarized in Additional file 1: Table S1.

**Fig. 2:**
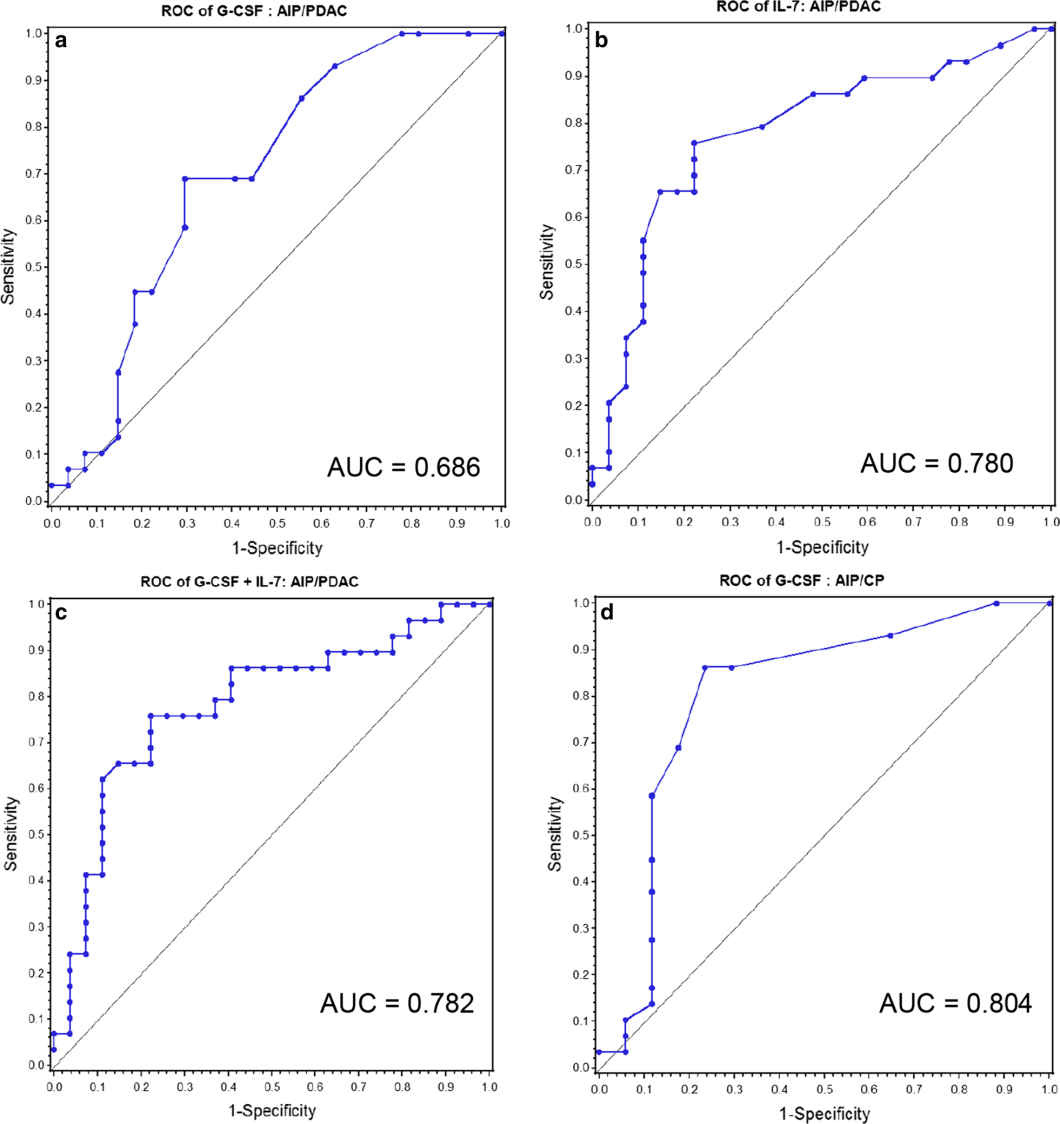
Receiver operator characteristic (ROC) analysis of serum IL-7 and G-CSF: **a**–**c** for differentiating patients with AIP from those with PDAC. **a** ROC curve for G-CSF. **b** ROC curve for IL-7, **c** ROC curve for combined index of G-CSF and IL-7. The respective areas under the* curve* (AUC) are 0.699 for G-CSF, 0.780 for IL-7, and 0.782 for the combination of both. **d** ROC curve for G-CSF for differentiating patients with AIP from those with CP (AUC = 0.804)

In order to evaluate the diagnostic utility of cytokines IL-7 and G-CSF for predicting AIP, a ROC curve analysis was carried out using the AIP, CP, and PDAC cytokine serum data. The obtained results are summarized in Table 3. IL-7 discriminated better than G-CSF AIP from PDAC (AUC of 0.780 vs 0.686). Combination of both IL-7 and G-CSF for differentiation only marginally improved the diagnostic value of the two markers (AUC = 0.782) as presented in Fig. 2; addition of IL-1β showed no further improvement (results not shown). Of note, the comparison between patients with CP and those with AIP, G-CSF alone performed better (AUC = 0.804) than the combination of both G-CSF and IL-7 (AUC = 0.787). Using G-CSF as reference curve the differences in the AUC reached significance in the AIP vs. CP comparisons (p = 0.0052) but not in the AIP vs PDAC using G-CSF as reference curve (p = 0.395).

**Table 3:** Diagnostic performance of serum IL-7, G-CSF, and in combination G-CSF + IL-7 as biomarkers for differential diagnosis

Marker		Sensitivity	Specificity	AUC
IL-7 (cut off 10.02 pg/ml)				
AIP vs PDAC		77.78	75.86	0.780
AIP vs CP		89.66	17.65	0.367
G-CSF (cut off 9.92 pg/ml)				
AIP vs PDAC		68.97	70.37	0.686
AIP vs CP		86.21	76.47	0.804
G-CSF + Il-7				
AIP vs PDAC		75.86	77.78	0.782
AIP vs CP		86.21	76.47	0.787

### Pancreatic tissue cytokine profiling

Similarly to the serum cytokine profiling, a tissue homogenate analysis was performed. The levels of cytokines in CP, PDAC, and both AIP forms were compared. Here, significantly higher concentrations (9.56 pg/mg) of G-CSF (p = 0.0138) were measured in AIP vs PDAC (3.44 pg/mg) and significantly lower levels were measured for IL-8 and TNF-α in the AIP group.

On the other hand, comparing the AIP and CP groups, AIP revealed significantly higher concentrations for IL-17 (p = 0.0120) and MIP-1β (p = 0.0326) and lower levels of TNF-α (p = 0.0038). The median concentration values and IQR are presented in Table 4.

**Table 4:** Cytokine levels in pancreatic tissue lysates from total AIP, CP and PDAC patients

Cytokine	Compared groups 1st group vs 2nd group	Median conc. (pg/mg) of 1st group/IQR/n	Median conc. (pg/mg) of 2nd group/IQR/n	p-value
IL-1β	AIP vs CP AIP vs PDAC	2.69/4.57/12 2.69/4.57/12	1.71/1.31/12 2.01/4.29/12	0.1332 0.3708
IL-6	AIP vs CP AIP vs PDAC	12.82/40.48/11 12.82/40.48/11	12.62/28.80/12 40.6/73.42/12	0.8055 0.2549
IL-7	AIP vs CP AIP vs PDAC	2.9/1.72/11 2.9/1.72/11	4.46/2.08/12 4.01/4.28/12	0.1090 0.4036
IL-8	AIP vs CP AIP vs PDAC	17.82/338.64/12 17.82/338.64/12	27.25/70.63/12 105.30/138.65/12	0.9770 *0.0304*
IL-10	AIP vs CP AIP vs PDAC	0.59/0.72/12 0.59/0.72/12	1.05/0.63/12 0.92/0.51/12	0.0732 0.2713
IL-13	AIP vs CP AIP vs PDAC	4.7/7.15/11 4.7/7.15/11	8.87/8.01/12 8.56/5.81/12	0.0694 0.2064
IL-17	AIP vs CP AIP vs PDAC	21.93/27.03/12 21.93/27.03/12	10.64/12.12/12 21.2/25.98/11	*0.0120* 0.4789
G-CSF	AIP vs CP AIP vs PDAC	9.56/11.67/12 9.56/11.67/12	1.98/8.64/12 3.44/7.40/12	0.1080 0.2790
IFN-γ	AIP vs CP AIP vs PDAC	7.02/27.31/12 7.02/27.31/12	25.7/17.67/12 19.50/20.66/12	0.0815 0.2167
MCP-1	AIP vs CP AIP vs PDAC	303.70/509.70/12 303.70/509.70/12	158.94/206.72/12 140.45/286.84/12	0.1260 0.2145
MIP-1 β	AIP vs CP AIP vs PDAC	224.20/354.68/12 224.20/354.68/12	90.17/132.30/12 112.90/277.46/12	*0.0326* 0.3708
TNF-α	AIP vs CP AIP vs PDAC	6.01/2.89/12 6.01/2.89/12	12.60/10.13/12 8.97/17.84/12	*0.0038* *0.0086*

Concentrations are expressed in pg/mg total protein

Italic values indicate significance of p value (p < 0.05)

*IQR* interquartile range, *n* number of observations

**Fig. 3:**
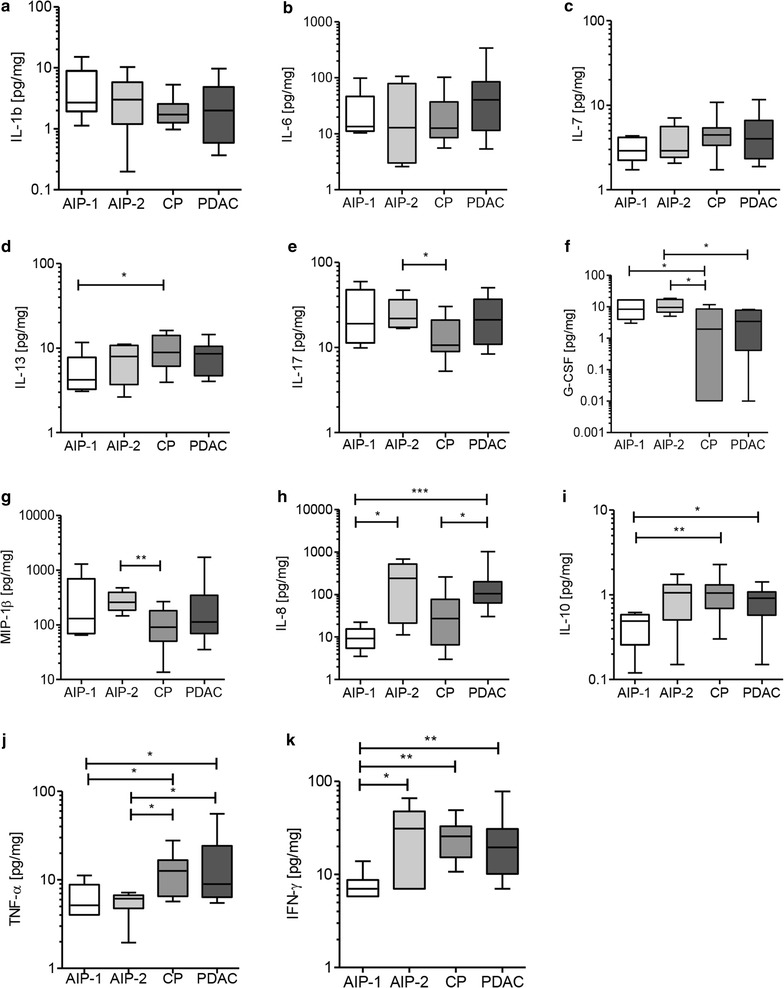
Comparison of the concentrations of different cytokines (**a**–**k**) in pancreatic tissue extracts. Values are presented in pg of cytokine per mg of total protein. Differences were considered statistically significant when the p value was less than 0.05 and are marked with an *asterisk*: *p < 0.05, **p < 0.01, and ***p < 0.001

Subsequently, all groups were separately compared either with subgroup AIP-1 or with AIP-2. Cytokines with interesting differences between the analyzed groups are presented in Fig. 3a–k and Additional file 2: Table S2. Of note, both AIP subtypes were distinguished from another by two cytokines IL-8 (p = 0.0152) and IFN-γ (p = 0.0376) showing higher levels in the AIP-2 subtype. IL-10 also showed slightly higher levels in AIP-2 (p = 0.0542).

## Discussion

The role of cytokines and growth factors in the development and progression of pancreatic inflammatory diseases including cancer has been intensively studied over recent decades [31]. In this study we investigated the ability of a variety of inflammatory mediators to detect and discriminated AIP from the pancreatic diseases CP and PDAC. The expression profile of 17 cytokines was evaluated in the serum and pancreatic tissue extracts in both AIP forms, either as one entity or segregated into AIP-1 and AIP-2, and compared with the levels found in CP and PDAC. Significant differences in serum of patients’ with AIP and PDAC were found for IL-1β, IL-7, and G-CSF, all with higher levels in AIP. G-CSF also revealed to be a good discriminator between both AIP, respectively their subtypes compared to CP and PDAC (Fig. 1f). This observation was also confirmed in the tissue extracts as shown in Fig. 3f.

We were able to demonstrate that IL-7 and G-CSF are valid serum markers for the differential diagnosis of AIP and PDAC. Combination of both cytokines revealed a slightly better differential diagnosis (AUC = 0.782). Likewise G-CSF turned out to be a good discriminatory marker between AIP and CP (AUC = 0.804)

G-CSF is a hematopoietic growth factor that induces proliferation of hematopoietic and cancer cells. It has been reported in different cancers including pancreatic cancer [32, 33]. A subtype of PDAC, which produce high levels of G-CSF (approx. 7%), is thought to attract high amounts of neutrophils, which can behave deleterious and are associated with a poor patients’ prognosis [34, 35]. In this study, we demonstrate for the first time the expression of G-CSF in AIP. It is known that different inflammatory stimuli such as IL-1β, IL-17, TNF-α, and lipopolysaccharide increase circulating levels of G-CSF that stimulate neutrophil production in the bone marrow [36, 37]. Interestingly, we found significantly higher expression of both IL-1β and G-CSF, which may be linked to a particular immune response linked to the AIP disease.

Interest in the functional effect of IL-7 in autoimmune diseases has grown in recent years due to the fact that enhanced IL-7 level fuels the proliferation of autoreactive T cells [38]. Given the role of IL-7 in T cell growth factor activity, it is not a surprise that increased levels of IL-7 have been reported in several autoimmune diseases such as multiple sclerosis [39], rheumatoid arthritis [40], type 1 diabetes [41], and systemic lupus erythematosus [42]. We also report here for the first time increased IL-7 serum levels in patients suffering from AIP.

In this study, MIP-1β and IL-17 allowed discrimination of AIP-2 from CP. It is known that elevated numbers of Th-17 cells and IL-17 levels, found in the periductal compartiments, are linked to the destruction of the pancreatic duct epithelium in AIP-2 [43]. Supporting the deleterious behavior of IL-17 it has been shown that IL-17 intensifies the effects of proinflammatory cytokines such as TNF-α, IL-1β, and IL-6 [44], thus orchestrating the formation of neutrophil extracellular traps (NETs) and the occlusion of pancreatic ducts leading to pancreatic inflammation [45].

Furthermore, higher expression of TNF-α was observed in our PDAC tissue lysates compared to AIP-1 and AIP-2 patients. It is well accepted that TNF-α induces pancreatic cancer cell proliferation [46]. TNF-α promotes the invasiveness of human pancreatic cancer cells and promotes tumor growth and metastasis in mice [31, 47].

As further finding, IL-8 and IFN-γ allowed discrimination between both AIP subtypes. These two cytokines were expressed in higher levels in the tissue of AIP-2 patients compared to these of AIP-1. IL-8 is a neutrophil-activating cytokine released from different cell types under inflammatory conditions [48]. IL-1 and TNF-α are the predominant stimuli that induce monocytes and macrophages to generate IL-8 [49]. Regarding IL-8 function, it is not surprising that its concentration is increased in the pancreatic tissue of patients suffering from AIP-2 since one of the hallmarks of AIP-2 is the presence of neutrophils in the pancreatic duct epithelia. Altogether, IL-1β, IL-7, IL-17, and G-CSF levels are increased in the serum of AIP-2 and IL-8 and IFN-γ levels in the pancreatic tissues of AIP-2 showing the importance of neutrophils in the pathophysiology of pancreatic inflammation.

## Conclusions

Clinically, it is of great relevance to distinguish PDAC from AIP. The significantly higher levels of IL-1β, IL-7, and G-CSF found in AIP-2 patients’ serum could be valuable markers in helping to distinguish it from PDAC. The results presented here show a high degree of reproducibility, particularly for G-CSF, providing reasons to prospectively evaluate this marker as a diagnostic tool. Currently, IgG4 is used as marker for AIP-1 but AIP-2 lacks a serological biomarker. Additionally, in AIP-1 cases where IgG4 levels are not elevated, the two cytokines IL-7 and G-CSF differentiated these AIP patients from PDAC. Applying a multiplex ELISA for IL-1β, IL-7, and G-CSF on patients’ serum would also allow AIP-2 to be distinguished from PDAC. Combining them with the recently identified gelatinases A and B and apolipoproteins Apo-AI and Apo-AII as discriminatory parameters [50–52] in a multiplex format, this could form the basis for a clinically applicable blood test, allowing reliable distinction between AIP and PDAC. This test would warrant immune-suppressive therapy without surgery for AIP patients and immediate surgical and chemotherapeutical treatment of PDAC patients. Wrong treatment of these modalities results in loss of precious time for cancer patients to obtain the right therapy and, on the other hand, causes AIP patients to undergo a huge operation, which is not necessary for this condition.

## Additional files

**Additional file 1: Table S1.** Comparison of cytokine levels in serum from: AIP-1, AIP-2, CP and PDAC patients.

**Additional file 2: Table S2.** Cytokine levels in pancreatic tissue lysates from AIP-1 and AIP-2 subtypes, CP and PDAC patients. Concentrations are expressed in pg/mg total protein.
