# Supplementary material for: Distinct pathophysiological cytokine profiles for discrimination between autoimmune pancreatitis, chronic pancreatitis, and pancreatic ductal adenocarcinoma
Source: J Transl Med. 2017 Jun 2;15:126. doi: 10.1186/s12967-017-1227-3 (PMC5457650; doi:10.1186/s12967-017-1227-3)
Supplement: Supplementary file 1 — Additional file 1: Table S1. Comparison of cytokine levels in serum from: AIP-1, AIP-2, CP and PDAC patients. [file 12967_2017_1227_MOESM1_ESM.docx]

**Supplementary Table S1:** Comparison of cytokine levels in serum from: AIP-1, AIP-2, CP and PDAC patients.

| Cytokine | Compared groups  1^st^ gr. vs 2^nd^gr. | Median conc. [pg/ml]  of 1^st^ group / IQR / n | Median conc. [pg/ml]  of 2^nd^ group / IQR / n | P-value |
| --- | --- | --- | --- | --- |
| IL-1β | AIP-1 vs AIP-2  AIP-1 vs PDAC  AIP-1 vs CP  AIP-2 vs PDAC  AIP-2 vs CP | 0.58 / 0.9 / 14  0.58 / 0.9 / 14  0.58 / 0.9 / 14  0.77 / 0.74 / 15  0.77 / 0.74 / 15 | 0.77 / 0.74 / 15  0.39 / 1.3 / 27  0.39 / 0.55 / 17  0.39 / 1.3 / 27  0.49 / 0.55 / 17 | 0.3176  0.1578  0.8876  **0.0217**  0.3619 |
| IL-6 | AIP-1 vs AIP-2  AIP-1 vs PDAC  AIP-1 vs CP  AIP-2 vs PDAC  AIP-2 vs CP | 12.02 / 16.36 / 13  12.02 / 16.36 / 13  12.02 / 16.36 / 13  14.23 / 74.6 / 15  14.23 / 74.6 / 15 | 14.23 / 74.6 / 15  10.89 / 22.42 / 27  1.44 / 25.63 / 17  10.89 / 22.42 / 27  1.44 / 25.63 / 17 | 0.2494  0.9195  0.7690  0.1937  **0.0361** |
| IL-7 | AIP-1 vs AIP-2  AIP-1 vs PDAC  AIP-1 vs CP  AIP-2 vs PDAC  AIP-2 vs CP | 16.38 / 11.13 / 14  16.38 / 11.13 / 14  16.38 / 11.13 / 14  12.90 / 6.14 / 15  12.90 / 6.14 / 15 | 12.90 / 6.14 / 15  8.27 / 3.65 / 27  8.72 / 13.04 / 17  8.27 / 3.65 / 27  8.72 / 13.04 / 17 | 0.1236  **0.0012**  0.0987  **0.0050**  0.3214 |
| IL-8 | AIP-1 vs AIP-2  AIP-1 vs PDAC  AIP-1 vs CP  AIP-2 vs PDAC  AIP-2 vs CP | 26.92 / 12.89 / 14  26.92 / 12.89 / 14  26.92 / 12.89 / 14  20.1 / 13.03 / 15  20.1 / 13.03 / 15 | 20.1 / 13.30 / 15  21.56 / 26.87 / 27  17.17 / 23.77 / 17  21.56 / 26.87 / 27  17.17 / 23.77 / 17 | 0.3257  0.9561  0.2110  0.2875  0.4613 |
| IL-10 | AIP-1 vs AIP-2  AIP-1 vs PDAC  AIP-1 vs CP  AIP-2 vs PDAC  AIP-2 vs CP | 8.07 / 20.75 / 13  8.07 / 20.75 / 13  8.07 / 20.75 / 13  2.38 / 5.55 / 15  2.38 / 5.55 / 15 | 2.38 / 5.55 / 15  4.09 / 9.59 / 21  0.4 / 12.10 / 13  4.09 / 9.59 / 21  0.4 / 12.10 / 13 | 0.3542  0.5827  0.1059  0.5958  0.1588 |
| IL-13 | AIP-1 vs AIP-2  AIP-1 vs PDAC  AIP-1 vs CP  AIP-2 vs PDAC  AIP-2 vs CP | 0.83 / 0.80 / 14  0.83 / 0.80 / 14  0.83 / 0.80 / 14  0.32 / 0.70 / 13  0.32 / 0.70 / 13 | 0.32 / 0.70 / 13  0.13 / 0.28 / 26  0.32 / 1.95 / 17  0.13 / 0.28 / 26  0.32 / 1.95 / 17 | 0.2537  **0.0162**  0.3156  0.3096  0.9327 |
| IL-17 | AIP-1 vs AIP-2  AIP-1 vs PDAC  AIP-1 vs CP  AIP-2 vs PDAC  AIP-2 vs CP | 3.75 / 16.26 / 14  3.75 / 16.26 / 14  3.75 / 16.26 / 14  11.3 / 20.34 / 15  11.3 / 20.34 / 15 | 11.3 / 20.34 / 15  4.32 / 19.32 / 27  0.03 / 10.35 / 17  4.32 / 19.32 / 27  0.03 / 10.35 / 17 | 0.1061  0.5536  0.6323  0.1524  **0.0377** |
| G-CSF | AIP-1 vs AIP-2  AIP-1 vs PDAC  AIP-1 vs CP  AIP-2 vs PDAC  AIP-2 vs CP | 14.23 / 16.85 / 14  14.23 / 16.85 / 14  14.23 / 16.85 / 14  14.23 / 14.31 / 15  14.23 / 14.31 / 15 | 14.23 / 14.31 / 15  5.23 / 13.82 / 27  1.47 / 3.94 / 17  8.44 / 13.82 / 27  5.23 / 3.94 / 17 | 0.8607  **0.0425**  **0.0034**  **0.0320**  **0.0084** |
| MCP-1 | AIP-1 vs AIP-2  AIP-1 vs PDAC  AIP-1 vs CP  AIP-2 vs PDAC  AIP-2 vs CP | 55.33 / 68.43 / 14  55.33 / 68.43 / 14  55.33 / 68.43 / 14  62.27 / 65.05 / 15  62.27 / 65.05 / 15 | 62.27 / 65.05 / 15  43.41 / 104.96 / 27  10.61 / 97.51 / 17  43.41 / 104.96 / 27  10.61 / 97.51 / 17 | 0.9825  0.4662  0.1529  0.5031  0.1125 |
| MIP-1β | AIP-1 vs AIP-2  AIP-1 vs PDAC  AIP-1 vs CP  AIP-2 vs PDAC  AIP-2 vs CP | 123.8 / 58.39 / 14  123.8 / 58.39 / 14  123.8 / 58.39 / 14  147.0 / 62.10 / 15  147.0 / 62.10 / 15 | 147.0 / 62.10 / 15  122.72 / 83.47 / 27  96.66 / 62.28 / 17  122.72 / 83.47 / 27  96.66 / 62.28 / 17 | 0.1692  0.5730  0.3934  0.4465  **0.0414** |

IQR : interquartile range, n: number of observations
