# Supplementary material for: Distinct pathophysiological cytokine profiles for discrimination between autoimmune pancreatitis, chronic pancreatitis, and pancreatic ductal adenocarcinoma
Source: J Transl Med. 2017 Jun 2;15:126. doi: 10.1186/s12967-017-1227-3 (PMC5457650; doi:10.1186/s12967-017-1227-3)
Supplement: Supplementary file 2 — Additional file 2: Table S2. Cytokine levels in pancreatic tissue lysates from AIP-1 and AIP-2 subtypes, CP and PDAC patients. Concentrations are expressed in pg/mg total protein. [file 12967_2017_1227_MOESM2_ESM.docx]

**Supplementary Table S2:** Cytokine levels in pancreatic tissue lysates from AIP-1 and AIP-2 subtypes, CP and PDAC patients. Concentrations are expressed in pg/mg total protein.

| Cytokine | Compared groups  1^st^ gr. vs 2^nd^gr. | Median conc. [pg/mg]  of 1^st^ group / IQR / n | Median conc. [pg/mg]  of 2^nd^ group / IQR / n | P-value |
| --- | --- | --- | --- | --- |
| IL-1β | AIP-1 vs AIP-2  AIP-1 vs CP  AIP-1 vs PDAC  AIP-2 vs CP  AIP-2 vs PDAC | 2.69 / 7.02 / 6  2.69 / 7.02 / 6  2.69 / 7.02 / 6  3.01 / 4.57 / 6  3.01 / 4.57 / 6 | 3.01 / 4.57 / 6  1.71 / 1.31 / 12  2.01 / 4.29 / 12  1.71 / 1.31 / 12  2.01 / 4.29 / 12 | 0.4098  0.1112  0.3736  0.4260  0.6065 |
| IL-6 | AIP-1 vs AIP-2  AIP-1 vs CP  AIP-1 vs PDAC  AIP-2 vs CP  AIP-2 vs PDAC | 13.46 / 35.48 / 6  13.46 / 35.48 / 6  13.46 / 35.48 / 6  12.82 / 75.58 / 5  12.82 / 75.58 / 5 | 12.82 / 75.58 / 5  12.62 / 28.80 / 12  40.60 / 73.42 / 12  12.62 / 28.80 / 12  40.60 / 73.42 / 12 | 0.9307  0.5740  0.3736  0.8744  0.3703 |
| IL-7 | AIP-1 vs AIP-2  AIP-1 vs CP  AIP-1 vs PDAC  AIP-2 vs CP  AIP-2 vs PDAC | 2.9 / 1.95 / 6  2.9 / 1.95 / 6  2.9 / 1.95 / 6  2.9 / 3.2 / 5  2.9 / 3.2 / 5 | 2.9 / 3.2 / 5  4.46 / 2.08 / 12  4.01 / 4.28 / 12  4.46 / 2.08 / 12  4.01 / 4.28 / 12 | 0.8535  0.0915  0.3229  0.4292  0.7916 |
| IL-8 | AIP-1 vs AIP-2  AIP-1 vs CP  AIP-1 vs PDAC  AIP-2 vs CP  AIP-2 vs PDAC | 9.24 / 10.04 / 6  9.24 / 10.04 / 6  9.24 / 10.04 / 6  241.30 / 501.34 / 6  241.30 / 501.34 / 6 | 241.30 / 501.34 / 6  27.25 / 70.63 / 12  105.30 / 138.65 / 12  27.25 / 70.63 / 12  105.30 / 138.65 / 12 | **0.0152**  0.1466  **0.0009**  0.1466  0.8883 |
| IL-10 | AIP-1 vs AIP-2  AIP-1 vs CP  AIP-1 vs PDAC  AIP-2 vs CP  AIP-2 vs PDAC | 0.49 / 0.33 / 6  0.49 / 0.33 / 6  0.49 / 0.33 / 6  1.06 / 0.82 / 6  1.06 / 0.82 / 6 | 1.06 / 0.82 / 6  1.05 / 0.63 / 12  0.92 / 0.51 / 12  1.05 / 0.63 / 12  0.92 / 0.51 / 12 | 0.0542  **0.0057**  **0.0192**  0.9253  0.6044 |
| IL-13 | AIP-1 vs AIP2  AIP-1 vs CP  AIP-1 vs PDAC  AIP-2 vs CP  AIP-2 vs PDAC | 4.23 / 4.54 / 6  4.23 / 4.54 / 6  4.23 / 4.54 / 6  7.97 / 7.09 / 5  7.97 / 7.09 / 5 | 7.97 / 7.09 / 5  8.87 / 8.01 / 12  8.56 / 5.81 / 12  8.87 / 8.01/ 12  8.56 / 5.81 / 12 | 0.9372  **0.0441**  0.0910  0.2061  0.4535 |
| IL-17 | AIP-1 vs AIP-2  AIP-1 vs CP  AIP-1 vs PDAC  AIP-2 vs CP  AIP-2 vs PDAC | 19.09 / 36.51 / 6  19.09 / 36.51 / 6  19.09 / 36.51 / 6  21.93 /1 9.19 / 6  21.93 / 19.19 / 6 | 21.93 / 19.19 / 6  10.64 / 12.12 / 12  21.2 / 25.98 / 11  10.64 / 12.12 / 12  21.2 / 25.98 / 11 | 0.6991  0.0678  0.6875  **0.0277**  0.4815 |
| G-CSF | AIP-1 vs AIP-2  AIP-1 vs CP  AIP-1 vs PDAC  AIP-2 vs CP  AIP-2 vs PDAC | 8.46 / 12.79 / 6  8.46 / 12.79 / 6  8.46 / 12.79 / 6  9.56 / 10.52 / 6  9.56 / 10.52 / 6 | 9.56 / 10.52 / 6  1.98 / 8.64 / 12  3.44 / 7.40 / 12  1.98 / 8.64 / 12  3.44 / 7.40 / 12 | 0.4665  **0.0387**  0.0824  **0.0311**  **0.0274** |
| IFN-γ | AIP-1 vs AIP-2  AIP-1 vs CP  AIP-1 vs PDAC  AIP-2 vs CP  AIP-2 vs PDAC | 7.02 / 2.93 / 6  7.02 / 2.93 / 6  7.02 / 2.93 / 6  31.06 / 40.62 / 6  31.06 / 40.62 / 6 | 31.06 / 40.65 / 6  25.7 / 17.67 / 12  19.50 / 20.66 / 12  25.7 / 17.67 / 12  19.50 / 20.66 / 12 | **0.0376**  **0.0016**  **0.0075**  0.7785  0.5385 |
| MCP-1 | AIP-1 vs AIP-2  AIP-1 vs CP  AIP-1 vs PDAC  AIP-2 vs CP  AIP-2 vs PDAC | 155.00 / 360.0 /6  155.00 / 360.0 /6  155.00 / 360.0 /6  532.20 / 880.29 / 6  532.20 / 880.29 / 6 | 532.21 / 880.29 / 6  158.90 / 206.72 / 12  140.50 / 286.84 / 12  158.90 / 206.72 / 12  140.50 / 286.84 / 12 | 0.5887  0.3736  0.4260  0.1223  0.2417 |
| MIP-1 β | AIP-1 vs AIP-2  AIP-1 vs CP  AIP-1 vs PDAC  AIP-2 vs CP  AIP-2 vs PDAC | 130.80 / 626.46 / 6  130.80 / 626.46 / 6  130.80 / 626.46 / 6  258.5 / 291.50 / 6  258.5 / 291.50 / 6 | 258.5 / 291.50 / 6  90.17 / 132.30 / 12  112.90 / 277.46 / 12  90.17 / 1.32.30 / 12  112.90 / 277.46 / 12 | 0.4848  0.4260  0.8883  **0.0087**  0.2061 |
| TNF-α | AIP-1 vs AIP-2  AIP-1 vs CP  AIP-1 vs PDAC  AIP-2 vs CP  AIP-2 vs PDAC | 5.17 / 4.79 / 6  5.17 / 4.79 / 6  5.17 / 4.79 / 6  6.12 / 1.93 / 6  6.12 / 1.93 / 6 | 6.12 / 1.93 / 6  12.60 / 10.13 / 12  8.97 / 17.84 / 12  12.60 / 10.13 / 12  8.97 / 17.84 / 12 | 0.9361  **0.0217**  **0.0350**  **0.0187**  **0.0348** |
